# Navigating possible endometriosis in primary care: a qualitative study of GP perspectives

**DOI:** 10.3399/BJGP.2021.0030

**Published:** 2021-08-03

**Authors:** Sharon Dixon, Abigail McNiven, Amelia Talbot, Lisa Hinton

**Affiliations:** Nuffield Department of Primary Care Health Sciences, University of Oxford, Oxford;; Nuffield Department of Primary Care Health Sciences, University of Oxford, Oxford;; Nuffield Department of Primary Care Health Sciences, University of Oxford, Oxford;; THIS Institute, Department of Public Health and Primary Care, University of Cambridge, Cambridge.

**Keywords:** dysmenorrhea, endometriosis, primary care, qualitative research, referral and consultation

## Abstract

**Background:**

Endometriosis affects approximately 6–10% of women, with well documented delays between initial presentation with symptoms and diagnosis. In England, women typically seek help first in primary care, making this setting pivotal in women’s pathways to diagnosis and treatment. English GP perspectives on managing possible endometriosis have not been previously reported.

**Aim:**

To explore what GPs identify as important considerations when caring for women with symptoms that raise the possibility of endometriosis.

**Design and setting:**

Qualitative study in English primary care.

**Method:**

Semi-structured scenario-based telephone interviews with 42 GPs from April 2019 to January 2020, based around a fictional scenario of a woman presenting to primary care with symptoms suggesting possible endometriosis. Interviews were thematically coded and analysed.

**Results:**

Managing possible endometriosis in primary care brings challenges. While knowledge and awareness were prerequisites for considering endometriosis, other important considerations were raised. Symptoms suggestive of endometriosis are non-specific, making endometriosis one possible consideration of many. GPs move through a diagnostic hierarchy to exclude sinister causes and utilise trials of treatment as both therapeutic interventions and diagnostic tools; processes which take time. An endometriosis label or diagnosis has advantages and risks. GPs reported sharing decisions about investigation and referral while holding women’s priorities as pivotal. These conversations were underpinned by their knowledge of uncertainties and unknowns, including the wide spectrum and unpredictability of endometriosis.

**Conclusion:**

GPs considerations are more complex than simply lacking awareness. The unknowns surrounding endometriosis matter to GPs. Further research and tailored resources for primary care, where women present with undifferentiated symptoms, are needed.

## INTRODUCTION

Endometriosis is the abnormal presence of endometrial tissue outside the uterus.^1^ Although the true prevalence is unknown, estimates suggest it affects approximately 6–10% of women of reproductive age.^2^^–^3 Endometriosis is seen in approximately 50% of women who present with sub-fertility and women who live with chronic pelvic pain.^2^^–^3 Endometriosis is definitively diagnosed in secondary care by surgical laparoscopy,^2^ a procedure associated with risks.^4^ But the route to diagnosis usually starts in primary care with initial symptom assessment and possible referral. GPs hold the care for women with endometriosis throughout their life-course, including during periods of involvement with specialist services and beyond.

Women face significant and well documented delays between their first visit to a doctor about symptoms and receiving a diagnosis of endometriosis.^5^^–^9 These delays can be associated with debilitating symptoms and worry^5^^,^^10^ and with increased healthcare costs.^10^^–^11 Retrospective studies demonstrate a time lag between women presenting to a GP with symptoms and being given a diagnosis of endometriosis.^7^^–^8 While these studies can reveal consulting patterns about symptoms before diagnosis that might suggest endometriosis,^7^^–^10 they do not provide insights into the deliberative processes in primary care that might help account for these documented delays; a knowledge gap this study sought to address.

It has been suggested that increased GP awareness and knowledge are needed to address these documented delays in diagnosis.^7^^,^^8^^,^^10^^,^^12^ The 2017 James Lind Alliance Priority Setting Partnership identified the need to understand how to effectively educate health professionals to reduce times to diagnosis and improve care for women with endometriosis as one of their top three endometriosis research priorities.^13^ By developing an understanding of how GPs approach the management of women with symptoms suggesting possible endometriosis, this study aimed to identify ways to support these care journeys in primary care. These insights can support the development of resources and education tailored for primary care and evolve beyond a simple call for increased awareness.

## METHOD

### Study design

A qualitative study was conducted using semi-structured telephone interviews based around a fictional scenario about a woman presenting to primary care with symptoms suggesting possible endometriosis (Box 1). A fictional scenario was used to create an arena for GPs to consider responses to possible issues they might face, an approach the authors have previously utilised.^14^

**Box 1.** Clinical scenario of patient with symptoms suggestive of endometriosis

*Clinical scenario:* Alice is 28 years old. She has made an appointment to talk to you because she is ‘fed up’ with having very painful periods. She has not seen a doctor about this before. She describes her periods as feeling ‘like knives stabbing from the inside’. She says that the pain has been so bad recently that she has had to take time off work. *Extensions to the clinical scenario:* Alice becomes tearful when she describes the impact of her period pain on her quality of life. Alice says she has looked online about her painful periods and is worried about what might be causing them. Would you do or approach anything differently if Alice was aged 17?

The scenario was co-developed with input from primary care (two GPs), a gynaecologist, and Patient and Public Involvement (PPI) advisers.[Table table3]

**Table table3:** How this fits in

There are documented time lags between women presenting to primary care with symptoms suggesting endometriosis and their receiving a diagnosis. It has been suggested that increasing GPs’ awareness will improve this situation. As GPs’ perspectives on these care journeys are not known, how best to educate health professionals to reduce delays in diagnosis is unclear. Even with awareness of the possibility of endometriosis, GPs’ accounts suggest that journeys are complex and can involve navigating significant uncertainties, including managing women whose symptoms are well controlled with primary care treatment, or who do not want to have referral or operative investigation. Primary care is well placed to support longitudinal care journeys for patients with possible or confirmed endometriosis. Evidence-based education and resources developed for primary care would support this role.

### Sampling and recruitment

With the support of the NIHR Local Clinical Research Network (LCRN) teams, GPs were recruited in five LCRN regions in England: Thames Valley and South Midlands, East Midlands, South West Peninsula, Greater Manchester, and North West Coast. These were selected aiming for a mixture of urban and rural settings, and areas with varied access to secondary and tertiary care and to support a sample as close to the real-world situation as possible. The sample comprised 42 GPs (19 male, 23 female). Recruitment was enhanced through snowballing.

### Data collection and analysis

Telephone interviews were conducted between April 2019 and January 2020. With consent, interviews were audiorecorded and transcribed verbatim. Field notes were taken which informed analysis if participants chose not to be recorded (*n* = 1 GP). A coding framework was iteratively developed in NVivo version 12 based on expected and emergent themes, and analysed thematically^15^ using mind-mapping techniques.^16^ All authors contributed to data analysis.

How the findings could be best represented and utilised was reviewed with: an expert stakeholder panel (2 gynaecologists and 2 GPs) and also with ten PPI collaborators with lived experience of endometriosis, primarily recruited via the Royal College of Obstetricians and Gynaecologists Women’s Voices Network. Based on the findings, some practical tips were co-developed with the PPI collaborators for GPs to consider when caring for women with possible endometriosis (Box 2).

**Box 2.** Tips that GPs could consider when caring for women with possible endometriosis, developed with women with lived experience of endometriosis

Recognise that women may have experienced painful symptoms for months or years *before* they make an appointment. Listen to the whole account of the woman’s experiences, including considering patterns of symptoms across more than one system (pelvis, bowels, bladder, fatigue, and so on), and that these might be dominant. Recognise that symptoms can be constant or cyclical. Ask women to compile or use symptom accounts or diaries to help spot symptom patterns and monitor changes, including in response to interventions. Support and continuity of care from GPs helps women. Arranging follow-up appointments can demonstrate your interest in helping. Do not make assumptions about women’s concerns or priorities (including about sexuality and reproductive intentions both now and in the future); ask them. Do not assume that distress is driving pain: it can be the other way round. It is good to respond to women’s concerns if they raise endometriosis. GPs should introduce the word and possibility if women do not. Trials of treatment require clear communication to be effective. GPs need to explain their thinking and ensure there is a robust shared follow-up plan including a clear timescale for review. To not do this risks women feeling ‘fobbed off’ and not coming back. Offer information and resources about endometriosis, even if this is only a possibility, as this can help women advocate for themselves. Keep an ‘open door’ and hold an ongoing advocacy role in primary care to help women navigate their care journeys, with recognition that endometriosis can be difficult to diagnose.

## RESULTS

The patient journey through primary care is used to present the analysis under the following thematic headings and subheadings:

- managing the initial consultation(s);
  — introducing the possibility of endometriosis;
- decisions about investigation and referral
  — the value of a diagnostic label
  — GP explanations for delays in diagnosis/the journey to diagnosis; and
- the long-term/enduring role(s) of primary care.

### Managing the initial consultation(s)

In the clinical scenario, ‘Alice’ presented with symptoms that had not been previously assessed, investigated, or diagnosed. For the GP, navigating the wide range of possible explanations for Alice’s symptoms was the first stage on the journey from symptom evaluation to considering possible diagnoses. This was a complex process of working through a clinical hierarchy, where possible ‘red flags’ needed to be evaluated and excluded before other diagnoses (including endometriosis) could be brought into active consideration:

*‘Always as a GP, we’re terrified of missing something horrible, so you think about red flags.’*

(GP30)

In parallel with considering possible explanations for Alice’s symptoms, GPs reflected that endometriosis can present in multiple ways, with a wide range of possible gynaecological and non-gynaecological symptoms. This clinical inconsistency and unpredictability presented GPs with added complexity during the initial evaluation:

*‘The symptoms can be very vague, from simple chronic pain, pelvic pain to pain related to periods, dysmenorrhoea to dyspareunia, bowel problems, chronic fatigue, other symptoms and consequences such as infertility, So the presentations are very different and that makes it challenging. And obviously you still have to consider a lot of diagnoses when you have a patient presenting in front of you.’*

(GP26)

GPs’ initial assessment included exploring any medications Alice had tried already and the impact of her symptoms on her emotional wellbeing and social functioning. This included exploring her expectations of the consultation, for example, symptom control, diagnosis, or addressing specific concerns such as fertility or fear of malignancy. Because endometriosis is not a diagnosis that can be made in primary care, the tests GPs ordered were predominantly and knowingly to rule out other potential causes.

#### Introducing the possibility of endometriosis

GPs reflected on how and when they might introduce endometriosis as a potential cause for Alice’s symptoms during the clinical journey from presentation with symptoms to investigations and/or treatment:

*‘If there’s something quite clear in the history that put endometriosis at the top of my list, then I might float it as a possible, yes. But I’d have to be quite sure that that was what’s going on.’*

(GP27)

Several GPs felt they were unlikely to raise endometriosis as a possible diagnosis during the first consultation. They described waiting to assess the impacts of treatments or until they judged that it was an appropriate moment, having developed a trusting relationship with Alice:

*‘It can help sometimes if you build up a bit of a relationship* […] *They might not want to tell you the first time they meet you that they’re actually having really painful sex, and they might be quite worried about that. Or they might not want to open up to you about that, but maybe once you’ve seen them once or twice, they might say.’*

(GP21)

Some GPs described their caution, concerned that by mentioning the word ‘endometriosis’ patients risked being adversely affected by researching and worrying about the potential consequences of endometriosis (for example, regarding fertility), when they might not have this diagnosis at all:

*‘You’re going to keep it generalised because you don’t want people to go away worrying that it’s X, Y, or Z, when actually at that point you might not know.’*

(GP33)

Some GPs worried that once endometriosis was mentioned, GPs would feel pressure to ‘follow through’ and refer to secondary care for a diagnosis to remove uncertainty:

*‘When I start that conversation, then I should be prepared to refer her for further investigations. Now, if I don’t do that, I think I would be in a very difficult situation having raised that diagnosis.’*

(GP41)

Other GPs felt that raising endometriosis as a possible diagnosis offered a doorway to an open conversation which could enable women to respond to emerging or evolving symptoms, or about potential risks and benefits of referral:

*‘I’m only mentioning it so you know I’d thought of it, and so that you know what to watch out for.’*

(GP42)

GPs described a range of strategies including introducing endometriosis as one possibility within a list of potential diagnoses or creating opportunities for the patient to raise endometriosis first. Sharing and discussing their thinking helped GPs ensure the patient understood what tests they were going for and why. Many felt that resources based on symptoms which offered balanced, accurate information that they could confidently signpost women to at this pre-diagnosis stage would help them with these conversations.

### Decisions about investigation and referral

GPs described how women’s priorities were paramount when navigating and discussing treatment, investigation and referral decisions. For example, if Alice wanted contraception then hormonal therapy would be appropriate to offer, but this would usually not be the case if she wanted help because of fertility concerns.

Several GPs described using trials of treatment (either before or in parallel with referral), usually with hormonal treatments (including contraceptive pills or the intrauterine system), as both a therapeutic intervention and one which could add information to the diagnostic process. To be effective in this way, these trials of treatment needed to be underpinned by continuity of care (ideally) and supported by effective communication both with the patient and any other clinicians who might see her at subsequent consultations:

*‘You have to really try and positively engage the patient to come back and really convince them that you want them to come back. Because you know if you give them a 3-month trial of treatment, it doesn’t help, and they think “‘Well nothing really happened last time, I’m not bothered, I’m going to leave it 6 months, and then I’m going to come back”, the years slip so quickly in that kind of timescale.’*

(GP39)

Referral was straightforward in some situations, such as if there were concerns about fertility (including established sub-fertility), severe symptoms that could not be adequately controlled with treatment available in primary care, symptoms suggesting extra-pelvic spread of endometriosis, and when the patient asked for referral for specialist care or a diagnosis.

Some GPs felt that all women with possible endometriosis should have referral offered. However, GPs also described how they approached caring for women whose symptoms were well controlled with primary care treatments or who said they did not want a referral to hospital. This would be a shared decision with the woman where she was offered choice:

*‘It is very much led by* […] *do they want to actually find out the underlying cause, or do they just want to treat the symptoms; it’s very much dependent on them.’*

(GP22)

GPs were mindful that diagnosis requires an invasive procedure in secondary care, and that laparoscopies have potential risks, including pelvic pain and adhesions:

*‘The only way you can really make it is with a laparoscopy and you don’t really want to put a woman through that unless you’ve reached the absolute end of the road.’*

(GP6)

Several GPs described experiences where referral into secondary care had resulted in the specialist electing to focus on symptom management, rather than undertaking any investigation to seek a diagnosis, which they could have done themselves in primary care. This could influence GPs expectations and approach to referral decisions:

*‘Sometimes they’re just discharged, aren’t they, with their oral contraceptive pill and I think they’re just a bit like, “Well my GP could have done that.” And I think sometimes GPs will feel “‘well can’t we just put them on the pill and then it’ll be what they were going to treat them with anyway’”*

(GP3)

#### The value of a diagnostic label

**Figure 1.**
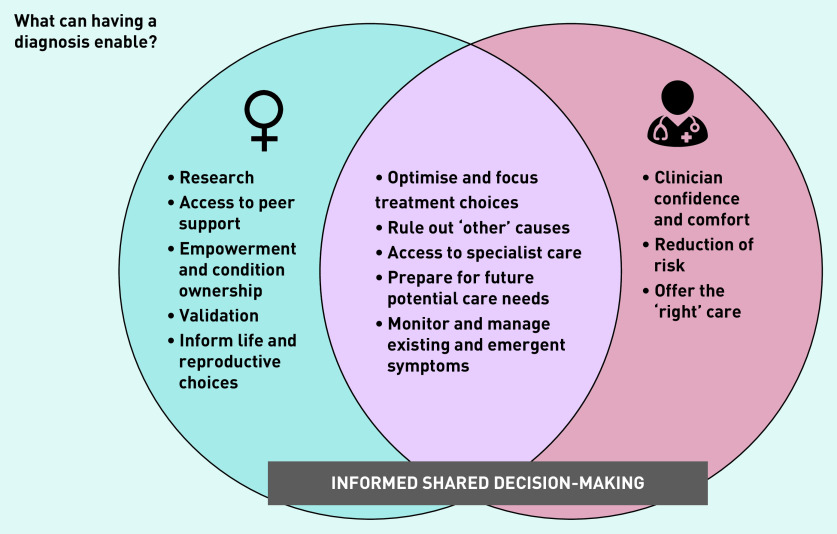
*GP perspectives on what a diagnosis can enable.*

GPs were largely supportive of the value of a diagnosis and identified its benefits for informing both current and future management (Figure 1). These included support for informed shared decision-making, offering access to specialist advice and treatment, and as a framework to support ongoing care needs, including new or emergent symptoms or concerns. However, the recognition of significant unknowns and uncertainties underpinned many GPs’ approach to the treatment and pursuit of a diagnosis. These included the variability of women’s experiences of endometriosis, the unpredictability of clinical sequelae, and whether a timely diagnosis would change treatment, or prevent or reduce future complications.

GPs were aware of the wide spectrum of symptoms and impacts of endometriosis, including the extent to which the endometriosis might be correlated, or not, with future fertility. Knowledge of this uncertainty shaped how they tailored their advice to individual patients. This included uncertainty when caring for women who had been incidentally diagnosed with endometriosis in the course of other investigations:

*‘...often you find spots of endometriosis and actually how much have you really helped someone in that situation? They’ve got a diagnosis, they’ve got worry, they know that they’ve got an increased risk of infertility possibility over the course of the data, it’s not necessarily applying quite as well to them if they’ve got a couple of spots, and it does, in the general person who, who has endometriosis.’*

(GP10)

When primary care treatment could not effectively manage Alice’s symptoms or concerns, this was considered clear grounds for onwards referral. However, if primary care treatment effectively managed her symptoms, whether further action was necessary or appropriate was an uncertainty reflected on by many GPs, and an area where they wished for guidance:

*‘Do I just give the pill, Am I reassured that even the pill will defer their endometriosis? I don’t know the answer to that really. So, if it* [endometriosis] *was busy doing its dastardly deed despite the pill, then...’*

(GP42)

While GPs said that they would ‘never stand in the way’ of a woman who wanted a diagnosis, some cautioned that having a diagnosis might not address all of her questions or concerns:

*‘Sometimes it’s a burden, well sometimes it’s a freedom* […] *People can be relieved to have a name, and I guess frustrated sometimes when there’s no name. But you know it goes both ways, doesn’t it? Because if they start looking for information, you know there’s a lot of devastating news out there. Because it can bring about more anxiety as well* […] *Although it’s a diagnosis, everyone is so different with their experience of endometriosis, it isn’t really that helpful, in a way.’*

(GP34)

In the original scenario, Alice was aged 28 years. GPs were then asked whether they would have different considerations if Alice was aged 17 when she first presented with dysmenorrhoea. GPs reflections on managing a teenaged Alice included the uncertainty about what ‘normal’ dysmenorrhoea is in adolescence, the balance between the benefits and risks of laparoscopy at this stage of life, and whether long-term outcomes would be influenced by an earlier diagnosis or treatment. GPs worried about holding responsibility for Alice’s fertility concerns, both in adolescence, and for what she may (or may not) want in her future:

*‘If it’s someone quite young, then at what point do you intervene and send them in for a laparoscopy? It might be that it can easily managed with the combined pill or something like that, the symptoms, but then you’re kind of worried in the back of your mind* […] *is that ultimately doing them any good? Is this endometriosis going to get worse while you’re doing that?’*

(GP27)

#### GP explanations for delays in diagnosis/the journey to diagnosis

**Figure 2.**
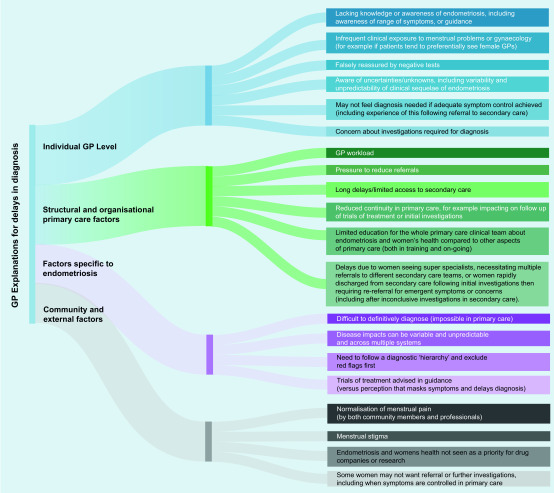
*GP explanations for delays in diagnosis.*

Potential explanations for the lag between presentation to primary care with symptoms and achieving diagnosis are presented in Figure 2. Some of these have already been discussed, including the need to follow a clinical hierarchy of diagnoses, the variability of the symptoms and impacts of endometriosis, concerns about laparoscopy, managing trials of treatment (as sanctioned in the National Institute for Health and Care Excellence [NICE] guidance),^17^ and supporting women who, at points in their care journey do not want referral (for example, if their symptoms are well controlled in primary care). Additional explanations for delays in diagnosis included GPs lack of awareness of or familiarity with endometriosis, lack of regular or focused education on women’s health and endometriosis, and challenges when negotiating the primary care/specialist interface.

### The long-term/enduring role(s) of primary care

Moving from initial presentation to primary care with symptoms to a diagnosis of endometriosis was experienced by GPs as a longitudinal journey which required iterative appraisal of both existing and evolving symptoms and priorities. GPs identified a long-term primary care role for women with both possible and confirmed endometriosis. This included supporting women through their diagnostic journey (including while they were also being cared for in secondary care) and managing emergent symptoms or concerns (for example, about fertility), and care needs in later life such as potential impacts of hormone replacement therapy on experiences of symptoms.

While having a confirmed diagnosis could be a helpful tool to underpin this long-term role, it was not always seen as essential:

*‘Actually, whether you call it endometriosis, to a certain extent what you do about it is maybe more important for the woman.’*

(GP1)

GPs identified that building rapport, developing trust, and having enduring primary care relationships were central to supporting this longitudinal care process, which could be facilitated by fostering and promoting continuity of care:

*‘You try to set up a bit of rapport beforehand because even though they’ll go to the gynaecologist, and maybe get their diagnosis, even then it seems that they kind of are given a treatment and discharged, and then they come back to primary care, and you sort of want to have established that rapport.’*

(GP3)

## DISCUSSION

### Summary

GPs’ descriptions of their assessment and support for women presenting to them with symptoms suggesting possible endometriosis reveal the complexity of this process. GPs recounted how symptom severity, personal impacts, and clinical needs associated with symptoms could vary widely, both between individual patients and throughout their life-course. While a diagnosis could be helpful for both women and clinicians, acquiring one required invasive testing, necessitating careful consideration of the balance between risks and benefits.

GPs sought to navigate this process by sharing decision-making with women, a process complicated by the variability of endometriosis and uncertainties about the long-term impact of interventions. GPs identified potential explanations for the documented delays in diagnosis, which offer opportunities for targeted interventions. Both the GPs interviewed in the study and the PPI collaborators emphasised the value of continuity of care and developing trusting primary care relationships to support these longitudinal journeys.

### Strengths and limitations

To the authors’ knowledge, this is the first study asking GPs working in England to offer their accounts for the process of evaluating and managing a woman with possible endometriosis. Presenting a scenario based on symptoms rather than a diagnosis offered insights into how GPs accounted for their decision-making, including how they explain delays in diagnosis. GPs with a range of experiences and from different locations were interviewed, and there was a balance between male and female GPs. Insights from the PPI collaborators helped the authors to use the research findings to co-create practical advice for GPs.

Participating GPs knew the study was about possible endometriosis, which might have influenced their responses or decision to participate; for example, if they had a preexisting interest in endometriosis. To ensure a sample which was as representative of the GP population as possible, GPs were recruited through the Clinical Research Network and not through women’s health networks. GPs were told what the study was about because the interview was not a test of knowledge, but an exploration of how GPs used their knowledge. The scenario was based around dysmenorrhoea, whereas endometriosis has many other potential presenting symptoms; however, this limitation was identified and discussed by GPs, which helped mitigate against potential bias.

This is a qualitative study, and while the findings are not necessarily generalisable, these GP perspectives undoubtedly offer valuable insights in an area where there has been scant previous research.

### Comparison with existing literature

Retrospective studies conducted using UK primary care databases demonstrate a time lag between presentation to primary care with symptoms and receiving a diagnosis of endometriosis.^7^^–^8 These studies offer valuable insights into the constellations of symptoms or patterns of consultation that might suggest possible endometriosis.^7^^–^10 Some conclude that increased GP awareness of these patterns of symptoms might reduce diagnostic delays.^7^^,^^8^^,^^10^^,^^12^^,^^18^

Qualitative studies exploring women’s experiences of endometriosis also call for GPs to have greater knowledge and awareness of endometriosis symptoms,^19^^–^22 and research highlights the importance of not ‘normalising’ menstrual pain.^5^^,^^23^^–^24 However, these studies arguably reflect partial samples.^25^

A systematic review and synthesis of qualitative research reporting women’s experiences of endometriosis included only participants recruited from specialist clinics or support groups and not directly from primary care.^19^

Endometriosis journeys in primary care typically start with presentation of undifferentiated symptoms which could indicate (or be identified as) a number of potential conditions.^7^^–^8^,^^10^ This study strengthens this observation and adds GPs’ accounts of their decision-making about the diagnostic journey, including decisions about referral and investigations. Ultrasound investigation is usually negative in endometriosis, and GPs are advised to be aware of this limitation to mitigate against delays;^7^ however, GPs in this study were aware of this likelihood and were predominantly and knowingly referring for ultrasound to exclude other causes, which could have accounted for the (as yet undiagnosed and undifferentiated) symptoms.

A questionnaire study with Dutch GPs found that they did not consider endometriosis as the diagnostic impression from the first consultation with symptoms.^18^ This potentially aligns with the accounts reported in this study of a progression through the clinical hierarchy, and contributes a possible explanation for this observation.

Focus groups with GPs, also in the Netherlands, reported that lack of knowledge and training about endometriosis was a potential barrier to referral, and also identified GPs’ uncertainty about the benefits of referral, notably for younger women.^26^ The study authors concluded that GPs seem to have a ‘low sense of urgency’ about diagnosing endometriosis, even when they are aware of it as a possible explanation for symptoms, whereas in the present study, GPs identified a number of significant advantages for themselves and their patients of having a diagnosis, albeit underpinned by known uncertainties. While the value of continuity of care and therapeutic relationships when supporting women with endometriosis has been previously documented in work with clinicians,^27^ no other studies were found describing how GPs characterise the process of shared decision-making in this clinical context.

That presentation with sub-fertility is likely to trigger a prompt referral by GPs has been previously described^7^ and is supported by what is reported here.

There is an existing narrative in the literature on endometriosis about suppression of symptoms with hormonal treatment being identified or constructed as a potential contributory factor for delays in diagnosis.^5^^,^^8^ Others have noted that this could represent adequate symptom suppression control with primary care treatment and identify the importance of ensuring a shared understanding of trials of treatment so that they are effectively followed up and do not result in delays.^7^ This aligns with the 2017 NICE guidance on endometriosis, which advises clinicians to: ‘*consider referring women to a gynaecology service for an ultrasound or gynaecology opinion’* if patients *‘have severe, persistent or recurrent symptoms of endometriosis, they have pelvic signs of endometriosis or initial management is not effective, not tolerated or is contraindicated.’* 17

Supporting trials of treatment within a framework of shared understanding about the possibility of endometriosis, with planned proactive follow up alongside nurturing continuity of care, was identified by the PPI collaborators as an important message for GPs (Box 2). Further guidance was not identified about what to do if treatment is effective in symptom management: a key area of uncertainty and reflection among the GPs in this study, which to the authors’ knowledge has not been previously documented. The GP accounts presented here suggest that some care pathways could be characterised as delays in diagnosis when viewed retrospectively from the point of diagnosis. However, when viewed contemporaneously within a process of responding to evolving care needs — for example, in the context of shared decision-making where there is effective symptom control — these may not necessarily represent avoidable or inappropriate delays.

### Implications for research and practice

Longitudinal studies starting prospectively from symptoms are needed to develop knowledge about the likelihood of important possible outcomes associated with dysmenorrhoea, including whether treatment has any prognostic impact on these. These studies should explore whether there is variability in management or outcomes by demographic variables that might suggest potential unmet or unexplored care needs.

Aligned with this, there is a pressing need for qualitative research with women and professionals to further develop an understanding of the processes of seeking and receiving care for menstrual symptoms to inform evidence-based, co-created, shared decision-making resources. This research would need to seek, hear, and represent voices and experiences across a range of care experiences, including age, location, ethnicity, language, care setting, and decision-making about referral and investigation.

Evidence is needed to inform the question of whether the most critical variable is time to diagnosis, or whether it may be more valuable to consider time to effective treatment and symptom control.

Improving GPs’ awareness of menstrual wellbeing and when to consider endometriosis is vital but needs to recognise the complexity of the process and the decisions being made.

GPs need evidence-based education and resources which are developed for primary care and which will resonate with their experiences, including supporting them in navigating uncertainties. Validated resources focused on symptoms rather than diagnoses could support this process. These GP perspectives on journeys to diagnosis, mirrored by women’s advice for GPs based on these, will support the development of these resources (Box 2). While increasing awareness of the value of diagnosis for both clinicians and patients is vital, this must be accompanied by adequate and timely access to specialist services, both at the point of diagnosis and beyond. To assume that all delays reflect poor care is overly simplistic.

As the PPI collaborators highlighted, by keeping minds (and doors) open, primary care is well placed to support longitudinal care and enduring advocacy for patients with suspected or confirmed endometriosis.
